# Sociodemographic, Attitudinal, and Behavioral Correlates of Using Nutrition, Weight Loss, and Fitness Websites: An Online Survey

**DOI:** 10.2196/10189

**Published:** 2019-04-04

**Authors:** Carlos A Almenara, Hana Machackova, David Smahel

**Affiliations:** 1 Faculty of Psychology Universidad Peruana de Ciencias Aplicadas Lima Peru; 2 Institute for Research on Children, Youth and Family (IVMDR) Department of Psychology Masaryk University Brno Czech Republic; 3 Department of Media Studies and Journalism Masaryk University Brno Czech Republic; 4 Faculty of Informatics Masaryk University Brno Czech Republic

**Keywords:** body image, compulsive behavior, diet, feeding and eating disorders, individuality, internet, user-computer interface, weight loss, social support

## Abstract

**Background:**

Nutrition, diet, and fitness are among the most searched health topics by internet users. Besides that, health-related internet users are diverse in their motivations and individual characteristics. However, little is known about the individual characteristics associated with the usage of nutrition, weight loss, and fitness websites.

**Objective:**

The aim of this study was to examine the individual factors associated with the usage of nutrition, weight loss, and fitness websites.

**Methods:**

An invitation to an online survey was published on 65 websites and discussion forums. In total, we employed data from 623 participants (aged 13 to 39 years, mean 24.11 [SD 5.26]). The measures included frequency of usage of nutrition, weight loss and fitness websites, excessive exercise, eating disorder symptomatology, internalization of the beauty ideal, weight status, and perceived online social support. Participants’ data were used as predictors in a base linear regression model.

**Results:**

The final model had an acceptable fit (χ^2^_10_ =14.1; *P*=.17; root mean square error of approximation=0.03; comparative fit index=0.99; Tucker-Lewis index=0.99). Positive associations were found between usage of (1) nutrition websites and being female, higher levels of excessive exercise, and perceived online social support; (2) weight loss websites and excessive exercise, internalization, being female, eating disorder symptomatology, and being overweight or obese; and (3) fitness websites and levels of excessive exercise, internalization, and frequency of internet use.

**Conclusions:**

The results highlighted the importance of individual differences in the usage of health-related websites.

## Introduction

### Background

Nutrition, diet, and fitness are among the most searched health topics by internet users [1]. The usage of websites covering those topics can be related to positive outcomes, such as a healthier diet or higher adherence to physical activity [2]. However, it can also be associated with negative outcomes such as unhealthy weight control behaviors [3,4]. This is worrisome considering that some internet users could accentuate their eating and weight-related problems (eg, eating disorders and obesity).

Notable efforts have been made to understand health-related internet users [2,5,6]. However, no study has empirically tested a group of already identified individual characteristics related with eating and weight-related problems and its link with the usage frequency of nutrition, weight loss, and fitness websites. This study is aimed to fill this gap because of the need for a better understanding of the psychological factors related to health behaviors, something crucial to develop successful interventions [7]. Moreover, by understanding which individual factors are associated with each of these websites, we can improve the design and communication of tailored health information. In other words, we will be able to provide more personalized information within nutrition, weight loss, and fitness websites to foster health-enhancing behaviors rather than health-compromising behaviors.

### Past Research

Health-related internet users are diverse in their motivations and individual characteristics [8]. From past research, we have identified a group of individual characteristics associated with the selection and usage frequency of nutrition, weight loss, and fitness websites in different studies: eating disorder symptomatology [4]; the extent of the internalization of the ideal body [9]; the levels of exercise [3]; social support [10]; body mass index (BMI); and sociodemographic characteristics, such as gender and age [2,11].

For instance, studies on internet usage regarding diet, weight loss, and fitness have found that as many as 85% of the users are female [12-14]. Notably, studies with young women have found that the use of Web-based weight loss information predicts disordered eating [4], whereas self-reported eating disorder also predicts the interest in diet and fitness websites [13]. Therefore, it is common to see individuals with disordered eating symptomatology joining online communities mostly to get tips and tricks for weight loss, as well as social support [15]. Similarly, overweight and particularly obese individuals who experience the stigma against obesity [16] commonly turn to the internet for weight loss solutions [17].

Internalization is another important individual factor. There is evidence that media spreads an ideal of beauty that people *internalize* and renders them more prone to inappropriately change their nutrition or physical activity habits, such as doing strenuous exercise to achieve the “ideal body” [18,19]. In this sense, it is suggested that individuals with high levels of internalization (ie, susceptible individuals) would gravitate to appearance-focused media content, such as websites focused on weight loss for appearance reasons. Following Perloff’s model, it should be noted that, rather than a one-way effect of media use on attitudes and behaviors, there would be a mutual reinforcement [9]. In other words, internalization not only accounts for the effect of media exposure [19] but also for a dispositional factor to subsequent media usage [20].

Although there is some evidence for the influence of online media on exercise and fitness [3], less is known about the association between excessive exercise and the usage of healthy lifestyle websites. For instance, a recent study exploring the characteristics of women who post travel images and women who post “fitspiration” images (ie, promoting a healthy lifestyle through fitness) found higher levels of compulsive exercise and disordered eating in the fitspiration group [21]. In contrast, another study found that the usage of mobile phone apps to keep track of meals or exercise routines, but not blogs or microblogging (eg, Instagram and Twitter) about nutrition and exercise, was associated with compulsive exercise [22].

Finally, large studies suggest that internet usage for health information is highly motivated by the opportunity to get advice from others [6]. For instance, a study of an online community of individuals with high levels of disordered eating found that the main motive to join the group was to get support and advice regarding weight loss [15]. Similarly, other studies suggest that some people turn to the internet to supplement professional medical advice, particularly when they are looking for advice on specific health issues or conditions [10,17]. Thus, online peer-to-peer health care reflects the importance of the online social support for adults’ health behaviors [7,10].

### This Study

Consequently, this study was aimed at exploring which individual factors relevant for the prevention of eating and weight-related problems are associated with the frequency of the usage of nutrition, weight loss, and fitness websites. We specifically examined the links with sociodemographic variables (ie, gender and age), eating disorder symptomatology, weight status, the tendency for excessive exercise, and the levels of internalization and perceived social support from website users. Upon this examination, we aimed to uncover which of the abovementioned factors could predict the usage of those websites. The results can be helpful in the design of tailored health messages for the prevention of eating and weight-related problems and the promotion of a healthy lifestyle.

## Methods

### Participants

The study utilized data from the visitors of websites focused on nutrition, weight loss, and exercise collected as part of a project on eating behaviors in the context of internet and technology use. It was approved by the Research Ethics Committee of Masaryk University. The data were collected through an open Web survey between May and October 2016. For participant recruitment, Czech websites oriented to nutrition, weight loss, and fitness were asked to publish an invitation for study participation. These included websites, Web magazines, blogs, social networking sites, and specific discussions forums which were all searched using keywords related to nutrition, weight loss, and fitness. In total, 307 different online platforms were asked to publish the invitation to the survey. The invitation was published on 65 websites and discussion forums (with a response rate of 21%). All the participants were informed about the purpose of the research, the estimated time needed to fill in the questionnaire, and the right to the questionnaire at any time were provided link to further information about the project; and were asked to provide informed consent by clicking on the link to the questionnaire. Participants were motivated by the chance to win 1 of 5 vouchers for an e-shop for the amount of 40 Euros each. From the original sample, which comprises 1002 respondents (age mean 24.82 (SD 6.85); 81.64% [818/1002] females), we excluded (1) participants aged 40 years and older because of low number of respondents in this age (3.59%, 36/1002) and to keep our sample more homogeneous in terms of age; (2) participants who did not provide a sufficient amount of data regarding their individual characteristics which were measured on the last page on the questionnaire (27.34%, 274/1002); and (3) participants who reported that the reason for the website visits was because of the health issues of someone else (therefore, possibly lacking the personal motivation connected to their own eating and health status) (5.49%, 55/1002). The latter reason for exclusion was indicated by the question “Do you visit the sites about nutrition or sports not for yourself, but mainly because you want to help with the nutrition or sport of another person (partner, child, parent, etc.)?” and the answer “Definitely applies”. This excluded 335 respondents in total. Moreover, we excluded respondents with occasional missing values (4.39%, 44/1002), yielding a final sample of 623 respondents aged 13 to 39 years (mean 24.11, SD 5.26), including 83.6% females (521/623). The majority of our respondents were Czech (91.7%, 571/623); 7.7% were Slovak (48/623); and 0.6% indicated other nationality (4/623). All of these respondents were internet users who go online “several times a week” (0.9%, 6/623), “almost daily” (9.9%, 62/623), and “daily” (88.9%, 554/623).

### Measures

#### Usage of Nutrition, Weight Loss, and Fitness Websites

The frequency of use was measured by the question “How often do you visit websites regarding nutrition, weight loss, or exercise and sport?” with answers on a 6-point scale with the response options as follows: 1 (Never), 2 (Almost never), 3 (Several times a month), 4 (Several times a week), 5 (Almost daily), and 6 (Daily). Respondents answered with regard to 3 types of websites, that is, those focused on *nutrition (eg, relating to specific diets and healthy meals)* (mean 4.38, SD 1.23); *weight loss (eg, diets or instructions on how to lose weight)* (mean 3.04, SD 1.45); and *fitness (regarding your exercise or sport, but not, eg, the results of professional athletes)* (mean 4.02, SD 1.39).

#### Gender and Age

Gender was coded in binary (0=males, 1=females), and age was also requested (mean 24.11, SD 5.26 years).

#### Excessive Exercise

A total of 5 items from the excessive exercise subscale from the Eating Pathology Symptoms Inventory Scales [23] answered on a 5-point scale ranging from 1 (Never) to 5 (Very often) were used. The scale was computed by averaging the items, with higher scores indicating greater tendency for excessive exercise (mean 3.05, SD 0.96; alpha=.87); factor analysis confirmed unidimensional structure, all loadings >.75.

#### Eating Disorder Symptomatology

The SCOFF screening tool [24] consisting of 5 items with Yes/No response options was used to identify a group of respondents potentially at risk of eating disorders. Those answering “Yes” on 2 or more items were classified as respondents at risk of having an eating disorder (47.2%, 294/623).

#### Weight Status

The respondents reported their current height (in centimeters) and weight (in kilograms), which were used to calculate their BMI (kg/m^2^) (mean 23.02, SD 4.24). Weight status data were then obtained using international cut-off points for adults [25] and adolescents [26]. Respondents were classified as either being *underweight* (6.4%, 40/623), having *normal weight* (70.3%, 438/623), and being *overweight* or *obese* (23.3%, 145/623).

#### Internalization of the Beauty Ideal

Respondents were asked *to what extent do the following statements apply to you in regards to these sites?*, with 3 items adapted from the Multidimensional Media Influence Scale [27]: *I am comparing my appearance with people on these sites*, *I am trying to look like the people on these sites*, and *the content on these sites inspire me in how to look attractive*. The items were answered on a 4-point scale ranging from 1 (*definitely does not apply*) to 4 (*definitely applies*). The scale was computed by averaging the items, with higher scores indicating higher internalization (mean 2.34, SD 0.90; alpha=.82); factor analysis confirmed unidimensional structure, all loadings >.83.

#### Perceived Online Social Support

Respondents were asked “To what extent do the following statements apply to you with regard to these sites?”, with 3 items adapted from the Online Social Support for Smokers Scale [28]: “I get advice and support here that I would not get elsewhere”, “It is encouraging to know that there are other people making similar efforts (with regard to nutrition or sport)”, and “I feel that other visitors (or authors) of sites are giving me support”. The items were answered on a 4-point scale ranging from 1 (Definitely does not apply) to 4 (Definitely applies). The scale was computed by averaging the items, with higher scores indicating higher perceived support (mean 2.80, SD 0.74; alpha=.72); factor analysis confirmed unidimensional structure, all loadings >.70.

### Statistical Analysis

To assess the links between the studied factors, we tested a base linear regression model with observed variables in which we predicted all 3 outcomes using MLR estimator in Mplus (version 7; Muthén & Muthén, Los Angeles, CA, USA). All paths between predictors and outcomes were allowed; weight status was included as a dummy variable with *normal weight* as reference category. In the second step, we constrained all nonsignificant (*P*<.05) paths to zero.

## Results

Results of the base linear regression model are shown below (Table 1).

**Figure 1:**
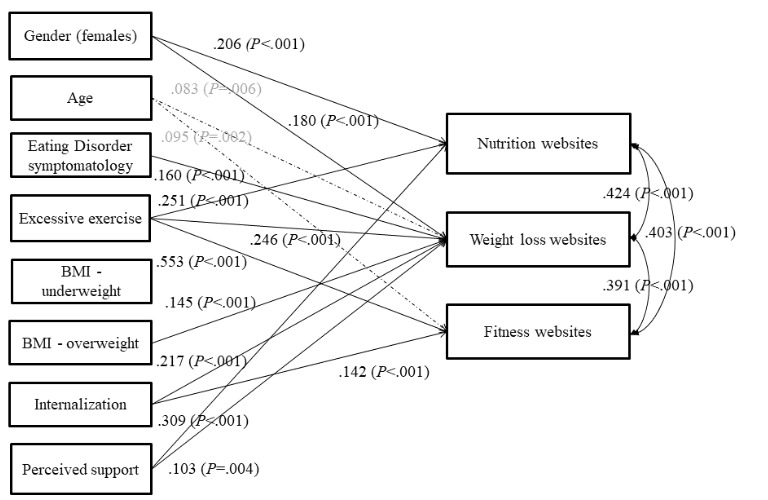
Final model predicting the frequency of the usage of nutrition, weight loss, and fitness websites. Standardized coefficients; effects with beta<.01 are dashed; the effect of perceived support on visits of fitness websites is not shown as it is not significant (beta=.056; *P*=.078). BMI: body mass index.

The final model (Figure 1) had an acceptable fit (χ^2^_10_=14.0, *P*=.17, root mean square error of approximation=0.03, comparative fit index=0.99, Tucker-Lewis index=0.99). The more frequent visits of nutrition websites were positively predicted by being female and having higher excessive exercise and higher perceived online social support. The visits of weight loss websites were predicted by being female and having higher excessive exercise, higher internalization, higher perceived online social support, eating disorder symptomatology, and being overweight (as opposed to having normal weight); the effect of age was significant but negligible (*beta*=.082). The visits of fitness-oriented websites were positively predicted only by higher excessive exercise and higher internalization; the effect of age was again significant but negligible (*beta*=.095), and the effect of perceived online social support was not significant in the final model (*P*=.078). Owing to the low number of respondents in the “underweight” category, we ran the analysis as well without this category; nevertheless, this supplemental analysis did not yield any substantially different results.

**Table 1:** Base regression model predicting the frequency of the usage of nutrition, weight loss, and fitness websites.

Variables	Nutrition^a^		Weight loss^b^		Fitness^c^	
	Beta	*P* value	Beta	*P* value	Beta	*P* value
Gender (females)	0.211	<.001	0.184	<.001	0.004	0.9
Age (years)	0.003	0.93	0.082	0.01	0.092	0.006
Eating disorder symptomatology	0.053	0.15	0.165	<.001	−.049	0.14
Excessive exercise	0.277	<.001	0.257	<.001	0.57	<.001
BMI^d^—underweight (vs normal)	0.066	0.06	−.003	0.94	0.032	0.35
BMI—overweight (vs normal)	0.026	0.49	0.152	<.001	0.011	0.73
Internalization	−.079	0.07	0.19	<.001	0.129	0.001
Perceived online social support	0.32	<.001	0.11	0.002	0.063	0.049

^a^Mean (SD) 4.38 (1.23); R^2^=.223.

^b^Mean (SD) 3.04(1.45); R^2^=.286.

^c^Mean (SD) 4.02(1.39); R^2^=.400.

^d^BMI: body mass index.

## Discussion

### Principal Findings

The aim of this study was to explore which individual factors relevant for the prevention of eating and weight-related problems were associated with the frequency of the usage of nutrition, weight loss, and fitness websites. Compared with previous studies that have examined separately those individual characteristics regarding the usage of those websites separately, this study was the first one to examine all those individual factors together regarding the usage of each of those 3 types of websites. Therefore, our study brings the opportunity to compare how an equal group of individual characteristics relate differently to the usage of nutrition, weight loss, and fitness websites. These results have several implications, as we show next.

Being female, having higher levels of excessive exercise, and perceived online social support were positively associated with the usage of nutrition websites. The usage of weight loss websites was positively associated with excessive exercise, internalization, being female, eating disorder symptomatology, and being overweight or obese, whereas its association with age and online social support was very weak. The frequency of fitness website usage was associated with the levels of excessive exercise, internalization (although this association was lower), and age (although this association was close to zero). In general terms, these findings contribute to a better understanding of the online health behavior of users of nutrition, weight loss, and fitness websites. Moreover, these results can contribute with future initiatives for the prevention of eating and weight-related problems through the internet. For example, our results suggested that providing social support in nutrition websites can be a venue to promote a healthy diet. We have discussed our results in more detail in the following lines.

### Usage of Nutrition Websites

Being female, having higher levels of excessive exercise, and perceived online social support were positively associated with the usage of nutrition websites. The lack of association with eating disorder symptomatology and internalization of the beauty ideal suggests that the usage of nutrition websites is motivated more for health orientation rather than body image concerns or eating pathology. This orientation toward a healthier lifestyle could also explain the association between exercise and the usage of nutrition websites, although this association remains largely unexplored and further research is recommended. In any case, there is a global trend among some young women, including Czech women, to pursue a healthier lifestyle by improving their diet, nutrition, and fitness [29]. In this sense, nutrition-oriented websites can represent an important and easily accessible source of information. Dietary information on the internet, such as on blogs and social media, is found to be very useful for internet users given that they can find recipe ideas for cooking and social support to have a healthier diet and/or to pursue a healthier lifestyle [3,30]. However, the use of nutrition websites to pursue a healthier diet raises questions about the accuracy, quality, and impact of the health-related nutrition information. For instance, health communication among online users may be contaminated by inaccurate health information and/or by health beliefs originated from the misinterpretation of the information that they find on the internet [31,32]. Thus, more research attention should be paid to the cognitive processing, evaluation, and selection of health information made by internet users while navigating through healthy lifestyle websites. Moreover, considering the association between social support and nutrition websites, several initiatives can be undertaken to promote healthy eating through these kinds of websites. For example, governments and international organizations can design and promote online social communities that foster appropriate nutrition, rather than solely providing relevant nutrition information.

### Usage of Weight Loss Websites

The usage of weight loss websites was positively associated with levels of exercise, internalization, being female, eating disorder symptomatology, and being overweight or obese. The usage of weight loss websites was also connected to age and online social support, although very weakly. Weight loss concerns are widespread among young women [33] and particularly among those with higher levels of internalization, those who are overweight, and those with disordered eating behavior [34,35]. Excessive exercise is frequently used for weight control purposes, and it is a common compensatory behavior among people with eating disorder symptomatology [36]. For overweight individuals, the internet is an easy-to-access source to find quick-fix weight loss solutions [17]. Therefore, taking all these individual factors together, it is possible that the underlying motivational factor for using these weight loss websites was body image concerns and particularly body weight concerns, which is a finding consistent with the literature [37]. Women who obtain weight loss information from the internet are more likely to exhibit unhealthy weight control behaviors [4]. Therefore, individuals’ concerns about body image, eating, and weight could have a bidirectional association with their own usage of weight loss websites, and their selective exposure (deliberately or not) to this kind of media information may shape their own media effects [20]. Thus, the selective exposure to weight loss information and its effect on disordered eating is definitively a venue for future research exploring the role of individual differences (eg, information processing of health claims regarding weight loss).

Finally, it is important to note that internet users with certain characteristics such as body or weight concerns, eating disorder symptomatology, and excessive exercise are more likely to upload inspirational content (ie, to promote weight loss) as well as fitspirational content [21]. In turn, through psychological mechanisms, such as observational learning [19], viewers of this content may feel inspired to pursue the “thin ideal body” and to adopt unhealthy weight control behaviors. Thus, media literacy interventions aimed at promoting a critical examination of media messages regarding weight loss may serve as a useful public health initiative to ameliorate the potential harmful effects of these kinds of messages [38]. Moreover, these initiatives should promote digital literacy skills that can serve as a countermeasure against the internalization of the thin ideal, for example, educating an internet user on how to utilize digital tools that block weight loss advertising while surfing these websites.

### Usage of Fitness Websites

The strongest association we found was between the levels of exercise and the frequency of fitness website usage, whereas its association with internalization was low and with age it was even lower. Following the selective exposure model [39], it could be suggested that individuals who engage in excessive exercise use fitness websites more frequently because the content on these websites is consistent with their beliefs and it can reinforce these beliefs [40]. In this sense, the role of internalization should be further explored. For instance, future research can examine how selective exposure to fitness websites among individuals with high levels of exercise may influence internalization as an enduring disposition or trait rather than as a state [35]. This approach would also contribute to further understanding the role of individual differences in the study of media effects [41].

### Differences Among Individual Factors Associated With the Usage of Nutrition, Weight loss, and Fitness Websites

Concerning sociodemographic factors, we only found a weak association between age and the usage of weight loss and fitness websites, which could be due to the characteristics of our sampling procedure because it was focused on a younger population (aged 13 to 39 years). On the contrary, being female was associated with the higher usage of nutrition websites and weight loss websites, which seems a consistent finding in recent surveys [2,5]. However, there was no link between gender and the frequency of use of fitness websites. Previous studies on internet use regarding diet, weight, and physical activity have found secular trends by gender, suggesting changes over time regarding gender differences in internet use [2]. Moreover, there are seasonal and geographical variations regarding physical activity and dieting [42,43] as well as in the frequency of internet searches for fitness and weight loss information [44]. Therefore, certain sociodemographic characteristics associated with the usage of nutrition, weight loss, and fitness websites are manifested differently over time and place. Future research should further investigate this dynamic aspect of health-related internet use and the sociodemographic characteristics associated with it. This approach is useful in the design of tailored health communication campaigns directed toward specific segments of the population (eg, specific clusters of young women within social networks).

With regard to individual factors, excessive exercise was moderately associated with all 3 types of websites. Excessive exercise is not a unique characteristic of individuals with eating disorders or those practicing sports. Indeed, fitness activities that are apparently healthy may become problematic when they lead to excessive exercise patterns due to individual factors such as personality traits (eg, perfectionism) [45]. Therefore, this is an interesting finding suggesting that the variance in exercise motivation [46], coupled with other individual factors such as internalization, determines the variance in the usage of lifestyle-related websites. This finding highlights the importance of using integrative approaches in the study of health behavior such as integrating motivational theories regarding exercise [46], health behavior [47], and internet use [48].

Our findings also revealed that internalization was positively associated with the usage of weight loss and fitness websites but not with the usage of nutrition websites. A recent meta-analytic review found a strong relationship between internalization and the use of appearance-related features in social networking sites [49]. Similarly, internalization is associated with higher levels of compulsive exercise [50]. Therefore, beauty ideal internalization seems to be a better predictor of the consumption of appearance-based online media (eg, fitness and weight loss websites) rather than health-oriented online media such as nutrition information. Furthermore, research is recommended to confirm this hypothesis.

Finally, perceived online social support was not associated with the usage of fitness websites but it was associated with the usage of nutrition websites and weight loss websites, although this latter association was weak. As mentioned above, nutrition websites usually provide information regarding specific diets and healthy meals as well as social interactions that internet users find very useful for their eating and dietary needs and goals [3,30]. Nevertheless, it should be noted that privacy attitudes are important in online health communication, particularly regarding the self-disclosure of body weight and weight loss concerns. For instance, anonymity is easier online compared with offline peer-to-peer communication, and studies have found that this anonymity in the online context provides an opportunity for shared self-disclosure of eating and weight loss concerns among members of online communities and blog users [51]. Therefore, the results of our sample could suggest that social support is particularly relevant regarding the usage of nutrition websites and to some extent the usage of weight loss websites but most probably not relevant regarding the usage of fitness websites.

In conclusion, the frequency in the use of nutrition, weight loss, and fitness websites is associated with a different combination of individual characteristics. Public health initiatives should consider such individual differences in the design of online strategies for the prevention of eating and weight-related problems.

### Limitations

Nevertheless, it is important to note that our study has some limitations. First, we explored the frequency of the usage of nutrition, weight loss, and fitness websites but not specific health behaviors related to these visits, such as the use of misinformation obtained from these websites. Thus, it would be beneficial to know more about how health-related information from these websites is actually used. In addition, the survey covered general patterns of visits to the 3 measured types of websites. However, it is probable that in some cases the visited websites could be defined by 2 or all 3 types. The data are also self-reported, a limitation which needs to be considered regarding the actual frequency of the visits (as compared with the recalled and reported one) as well as with eating disorder symptomatology, body weight, and height. Our sample is also limited in terms of generalizability, considering, for instance, that women were overrepresented and underweight individuals were underrepresented. Finally, the cross-sectional and correlational nature of our data precludes causal interpretations.

Although our study has limitations, it also has strengths. For instance, we asked participants about their frequency of use of specific websites rather than just asking them about online health information seeking. Moreover, this was a population-based survey that included diverse participants of both genders rather than a specific sample such as female college students. Finally, our results have important implications, particularly for the prevention of eating and weight-related problems such as eating disorders and obesity. Public health policies can be implemented to help with the personalization of Web content targeting individuals with a higher risk of developing eating and weight-related problems. For example, these policies can help to disseminate tailored public health messages (eg, about a healthy diet rather than weight loss), targeting specific users of lifestyle websites (eg, those with high internalization).

## Abbreviations

BMI: body mass index

## References

[1] Fox S (2005). Pew Research Center.

[2] McCully SN, Don BP, Updegraff JA (2013). Using the internet to help with diet, weight, and physical activity: results from the Health Information National Trends Survey (HINTS). J Med Internet Res.

[3] Vaterlaus JM, Patten EV, Roche C, Young JA (2015). #Gettinghealthy: the perceived influence of social media on young adult health behaviors. Comput Human Behav.

[4] Laz TH, Berenson AB (2011). Association of web-based weight loss information use with weight reduction behaviors in adolescent women. J Adolesc Health.

[5] Pollard CM, Pulker CE, Meng X, Kerr DA, Scott JA (2015). Who uses the internet as a source of nutrition and dietary information? An Australian population perspective. J Med Internet Res.

[6] Torrent-Sellens J, Díaz-Chao Á, Soler-Ramos I, Saigí-Rubió F (2016). Modelling and predicting eHealth usage in Europe: a multidimensional approach from an online survey of 13,000 European Union internet users. J Med Internet Res.

[7] Hoppmann C, Lay JC, Shayanfar S, Diehl M, Hooker K, Sliwinski MJ (2014). Intraindividual variability in the context of adults' health behavior. Handbook of Intraindividual Variability Across the Life-Span.

[8] Dutta-Bergman M, Murero M, Rice RE (2006). Media use theory and internet use for health care. The Internet and Health Care: Theory, Research, and Practice.

[9] Perloff RM (2014). Social media effects on young women's body image concerns: theoretical perspectives and an agenda for research. Sex Roles.

[10] Fox S (2011). Pew Research Center.

[11] Vaterlaus JM, Jones RM, Patten EV, Cook JL (2015). An exploratory study of time spent with interactive technology and body mass among young adults. Comput Human Behav.

[12] Wangberg SC, Sørensen T, Andreassen HK (2015). Using the Internet to support exercise and diet: a stratified Norwegian survey. Med 2 0.

[13] Carrotte ER, Vella AM, Lim MS (2015). Predictors of "Liking" three types of health and fitness-related content on social media: a cross-sectional study. J Med Internet Res.

[14] Kontos E, Blake KD, Chou WY, Prestin A (2014). Predictors of eHealth usage: insights on the digital divide from the Health Information National Trends Survey 2012. J Med Internet Res.

[15] Rodgers RF, Skowron S, Chabrol H (2012). Disordered eating and group membership among members of a pro-anorexic online community. Eur Eat Disord Rev.

[16] Puhl RM, Peterson JL, Corrigan PW (2014). The nature, consequences,public health implications of obesity stigma. The Stigma of Disease and Disability: Understanding Causes and Overcoming Injustices.

[17] Lewis S, Thomas SL, Blood RW, Castle D, Hyde J, Komesaroff PA (2011). 'I'm searching for solutions': why are obese individuals turning to the internet for help and support with 'being fat'?. Health Expect.

[18] Turner JS (2014). Negotiating a media effects model: addendums and adjustments to Perloff's framework for social media's impact on body image concerns. Sex Roles.

[19] Levine MP, Harrison K, Bryant J, Oliver MB (2009). Effects of media on eating disorders and body image. Media Effects: Advances in Theory and Research. 3rd edition.

[20] Valkenburg PM, Peter J, Walther JB (2016). Media effects: theory and research. Annu Rev Psychol.

[21] Holland G, Tiggemann M (2017). "Strong beats skinny every time": disordered eating and compulsive exercise in women who post fitspiration on Instagram. Int J Eat Disord.

[22] Hefner V, Dorros SM, Jourdain N, Liu C, Tortomasi A, Greene MP, Brandom C, Ellet M, Bowles N (2016). Mobile exercising and tweeting the pounds away: the use of digital applications and microblogging and their association with disordered eating and compulsive exercise. Cogent Soc Sci.

[23] Forbush KT, Wildes JE, Pollack LO, Dunbar D, Luo J, Patterson K, Petruzzi L, Pollpeter M, Miller H, Stone A, Bright A, Watson D (2013). Development and validation of the Eating Pathology Symptoms Inventory (EPSI). Psychol Assess.

[24] Hill LS, Reid F, Morgan JF, Lacey JH (2010). SCOFF, the development of an eating disorder screening questionnaire. Int J Eat Disord.

[25] World Health Organization (2000). Obesity: Preventing and managing the global epidemic.

[26] Cole TJ, Lobstein T (2012). Extended international (IOTF) body mass index cut-offs for thinness, overweight and obesity. Pediatr Obes.

[27] Cusumano DL, Thompson JK (2001). Media influence and body image in 8-11-year-old boys and girls: a preliminary report on the multidimensional media influence scale. Int J Eat Disord.

[28] Graham AL, Papandonatos GD, Kang H, Moreno JL, Abrams DB (2011). Development and validation of the online social support for smokers scale. J Med Internet Res.

[29] (2015). Euromonitor International.

[30] Bissonnette-Maheux V, Provencher V, Lapointe A, Dugrenier M, Dumas AA, Pluye P, Straus S, Gagnon M, Desroches S (2015). Exploring women's beliefs and perceptions about healthy eating blogs: a qualitative study. J Med Internet Res.

[31] Bonnar-Kidd KK, Black DR, Mattson M, Coster D (2009). Online physical activity information: will typical users find quality information?. Health Commun.

[32] Mitu B, Marinescu V, Mitu B (2016). Health in the digital era: searching health information online. The Power of the Media in Health Communication.

[33] Julia C, Péneau S, Andreeva VA, Méjean C, Fezeu L, Galan P, Hercberg S (2014). Weight-loss strategies used by the general population: how are they perceived?. PLoS One.

[34] Neumark-Sztainer DR (2015). Higher weight status and restrictive eating disorders: an overlooked concern. J Adolesc Health.

[35] Karazsia BT, van Dulmen HM, Wong K, Crowther JH (2013). Thinking meta-theoretically about the role of internalization in the development of body dissatisfaction and body change behaviors. Body Image.

[36] Fernandez-del-Valle M, Robert-McComb JJ, Norman RL, Zumwalt M (2014). Screening tools for excessive exercise in the active female. The Active Female: Health Issues Throughout the Lifespan.

[37] Rodgers RF, Melioli T (2016). The relationship between body image concerns, eating disorders and internet use, Part I: a review of empirical support. Adolesc Res Rev.

[38] Bergsma L, Ferris E, Preedy VR, Watson RR, Martin CR (2011). The impact of health-promoting media-literacy education on nutrition and diet behavior. Handbook of Behavior, Food and Nutrition.

[39] Knobloch-Westerwick S, Sarge MA (2015). Impacts of exemplification and efficacy as characteristics of an online weight-loss message on selective exposure and subsequent weight-loss behavior. Commun Res.

[40] Slater MD (2007). Reinforcing spirals: the mutual influence of media selectivity and media effects and their impact on individual behavior and social identity. Commun Theory.

[41] Oliver MB, Krakowiak KM, Bryant J, Zillmann D (2009). Individual differences in media effects. Media Effects: Advances in Theory and Research. 3rd edition.

[42] Gracia-Marco L, Ortega FB, Ruiz JR, Williams CA, Hagströmer M, Manios Y, Kafatos A, Béghin L, Polito A, De Henauw S, Valtueña J, Widhalm K, Molnar D, Alexy U, Moreno LA, Sjöström M, Helena Study Group (2013). Seasonal variation in physical activity and sedentary time in different European regions. The HELENA study. J Sports Sci.

[43] Markey PM, Markey CN (2013). Annual variation in internet keyword searches: linking dieting interest to obesity and negative health outcomes. J Health Psychol.

[44] Madden KM (2017). The seasonal periodicity of healthy contemplations about exercise and weight loss: ecological correlational study. JMIR Public Health Surveill.

[45] Cook B, Hausenblas H, Freimuth M, Brewerton T, Dennis AB (2014). Exercise addiction and compulsive exercising: relationship to eating disorders, substance use disorders, and addictive disorders. Eating Disorders, Addictions and Substance Use Disorders: Research, Clinical and Treatment Perspectives.

[46] Hagger MS, Ryan RM (2012). Advances in motivation in exercise and physical activity. The Oxford Handbook of Human Motivation.

[47] Dunsmore S, Goodson P (2013). Motivation for healthy behavior: a review of health promotion research. Am J Health Educ.

[48] Amiel T, Sargent SL (2004). Individual differences in internet usage motives. Comput Hum Behav.

[49] Mingoia J, Hutchinson AD, Wilson C, Gleaves DH (2017). The relationship between social networking site use and the internalization of a thin ideal in females: a meta-analytic review. Front Psychol.

[50] Homan K (2010). Athletic-ideal and thin-ideal internalization as prospective predictors of body dissatisfaction, dieting, and compulsive exercise. Body Image.

[51] Leggatt-Cook C, Chamberlain K (2012). Blogging for weight loss: personal accountability, writing selves, and the weight-loss blogosphere. Sociol Health Illn.

